# Identification of loci conferring resistance to 4 foliar diseases of maize

**DOI:** 10.1093/g3journal/jkad275

**Published:** 2023-12-05

**Authors:** Yuting Qiu, Pragya Adhikari, Peter Balint-Kurti, Tiffany Jamann

**Affiliations:** Department of Crop Sciences, University of Illinois Urbana-Champaign, Urbana, IL 61801, USA; Department of Crop Sciences, University of Illinois Urbana-Champaign, Urbana, IL 61801, USA; Department of Entomology and Plant Pathology, North Carolina State University, Box 7616, Raleigh, NC 27695, USA; Plant Science Research Unit, USDA-ARS, Raleigh, NC 27695, USA; Department of Crop Sciences, University of Illinois Urbana-Champaign, Urbana, IL 61801, USA

**Keywords:** multiple disease resistance, maize, multivariate analysis, joint stepwise regression

## Abstract

Foliar diseases of maize are among the most important diseases of maize worldwide. This study focused on 4 major foliar diseases of maize: Goss's wilt, gray leaf spot, northern corn leaf blight, and southern corn leaf blight. QTL mapping for resistance to Goss’s wilt was conducted in 4 disease resistance introgression line populations with Oh7B as the common recurrent parent and Ki3, NC262, NC304, and NC344 as recurrent donor parents. Mapping results for Goss’s wilt resistance were combined with previous studies for gray leaf spot, northern corn leaf blight, and southern corn leaf blight resistance in the same 4 populations. We conducted (1) individual linkage mapping analysis to identify QTL specific to each disease and population; (2) Mahalanobis distance analysis to identify putative multiple disease resistance regions for each population; and 3) joint linkage mapping to identify QTL across the 4 populations for each disease. We identified 3 lines that were resistant to all 4 diseases. We mapped 13 Goss’s wilt QTLs in the individual populations and an additional 6 using joint linkage mapping. All Goss’s wilt QTL had small effects, confirming that resistance to Goss’s wilt is highly quantitative. We report several potentially important chromosomal bins associated with multiple disease resistance including 1.02, 1.03, 3.04, 4.06, 4.08, and 9.03. Together, these findings indicate that disease QTL distribution is not random and that there are locations in the genome that confer resistance to multiple diseases. Furthermore, resistance to bacterial and fungal diseases is not entirely distinct, and we identified lines resistant to both fungi and bacteria, as well as loci that confer resistance to both bacterial and fungal diseases.

## Teaser

Multiple disease resistance is of interest to plant breeders as it can accelerate breeding for disease resistance. Qiu *et al*. analyze four maize introgression line populations to identify regions conferring resistance to multiple fungal diseases and the bacterial disease Goss’s wilt. They report several regions associated with resistance to multiple diseases, as well as several small effect QTL for Goss’s wilt resistance. Their data suggest that resistance to Goss’s wilt is highly quantitative.

## Introduction

Crops need to fend off many pathogens throughout the growing season and every year, there are major losses due to plant diseases. Several diseases can cause yield impacts, but individual diseases often vary from year to year in severity and importance. Multiple disease resistance (MDR), defined as host resistance to 2 or more diseases (Nene 1988), is thus of importance. The genetic architecture of MDR varies; it can be conferred either by a single gene or many genes across the genome (Wiesner-Hanks and Nelson 2016). In any given year, several diseases cause major losses and, thus, MDR is important for disease management, preventing crop losses and achieving yield stability. Although the introgression of many separate resistance loci for multiple diseases into a single variety is possible, the process can be time consuming and expensive (Singh *et al.* 2015). Therefore, pleiotropic MDR alleles are highly desirable in breeding programs.

Maize is a staple crop and important for human consumption, but, on average, 22.5% of the annual global yield of maize is lost due to pathogens and pests (Savary *et al.* 2019). Climate change has already begun to shift the range of maize diseases, in particular by favoring maize foliar diseases through improved pathogen overwintering in maize residue (Broders *et al.* 2022); consequently, the need for durable MDR will become especially crucial (Miedaner and Juroszek 2021). Foliar diseases can reduce the effective photosynthetic area of the plant, reducing yield. Foliar diseases caused the largest estimated maize yield losses in the northern United States and Ontario, compared to root and seedling rots, leaf blights, stalk rots, ear rots, and mycotoxin contamination, according to surveys from 2012 through 2019 (Mueller *et al.* 2016, 2020). Among the important maize foliar diseases are the bacterial disease Goss's wilt (GW) and leaf blight caused by *Clavibacter nebraskensis*, and 3 fungal diseases; gray leaf spot (GLS) caused by *Cercospora zeae maydis*, northern corn leaf blight (NCLB) caused by *Exserohilum turcicum*, and southern corn leaf blight (SCLB) caused by *Bipolaris maydis*.

GW was first detected in 1969 in south central Nebraska, re-emerged in 2006 and since then, the disease has been a widespread concern across the United States and Canada (Vidaver and Mandel 1974; Jackson *et al.* 2007; Harding *et al.* 2018; Mueller *et al.* 2020; Webster *et al.* 2020). In inoculated trials, GW caused up to a 40% yield loss, and in severe infections ears may not develop (Pataky 1988; Carson 1991; Cooper *et al.* 2019). For every 1% increase in R1 (silking stage) disease severity on a susceptible hybrid, yield was reduced by 117 kg/ha (1.9 bu/acre) (Bauske and Friskop 2021). Currently, there is no effective chemical control available for the disease and very few options are available to growers other than genetic resistance, so host resistance is the primary method used to manage GW (Mehl *et al.* 2015; Osdaghi *et al.* 2023). Methods of breeding for resistance and sources of resistance (Carson 1991; Cooper *et al.* 2019; Jindal *et al.* 2019; Mehl *et al.* 2021), as well as regions and genes associated with resistance have been investigated (Schaefer and Bernardo 2013; Singh *et al.* 2016; Cooper *et al.* 2018; Hu *et al.* 2018; Owusu *et al.* 2019; Singh *et al.* 2019; Qiu, Cooper *et al.* 2020; Li *et al.* 2022; Hao *et al.* 2023). GW, along with NCLB and GLS, was among the most destructive diseases from 2012 to 2019 in the northern United States and Ontario (Mueller *et al.* 2016, 2020).

A previous study (Wisser *et al.* 2006) documented evidence for the nonrandom distribution of maize disease QTLs, suggesting the existence of loci associated with resistance to more than 1 disease. For instance, McMullen and Simcox (1995) identified clusters of QTLs in chromosomal bins 3.04 and 6.01 that were associated with Fusarium stalk rot and European corn borer and single factor genes for resistance to common rust, wheat streak mosaic virus, and maize mosaic virus. Several genes conferring MDR, implicating diverse mechanisms, have been reviewed by Wiesner-Hanks and Nelson (2016) and Gou *et al*. (2023). In maize, an MDR gene *ZmCCoAOMT2*, which encodes a caffeoyl-CoA *O*-methyltransferase, confers resistance to SCLB and GLS (Yang *et al.* 2017). Additionally, *ZmMM1*, a MYB transcription repressor, confers resistance to NCLB, GLS, and southern corn rust (Wang *et al.* 2021) and the *ZmFLR* genes are involved in resistance to NCLB, SCLB, northern leaf spot, and anthracnose stalk rot (Yu *et al.* 2022).

Near-isogenic lines are an excellent genetic resource for dissecting MDR. Previously, Wisser *et al.* (2011) screened a diversity panel for NCLB, SCLB, and GLS and identified lines that were resistant or susceptible to NCLB, SCLB, and GLS. Using these lines, Lopez-Zuniga *et al.* (2019) created a set of BC_3_F_4:5_ chromosome segment substitution line (CSSL) populations, referred to as the disease resistance introgression line (DRIL) populations. There are 8 DRIL populations that consist of lines containing segments from 4 MDR donor lines introgressed into the genetic background of 1 of 2 multiple disease susceptible (MDS) lines. These populations were developed to better capture the effects of resistance loci in a susceptible background and to identify MDR near-isogenic lines (NILs) to use in subsequent confirmation studies (Lopez-Zuniga *et al.* 2019; Martins *et al.* 2019). We noted that the MDR donor lines for these populations were also resistant to the bacterial diseases GW and bacterial leaf streak (BLS), while the MDS recurrent parents were susceptible (Cooper *et al.* 2019; Qiu, Kaiser *et al.* 2020). Therefore, in addition to their use to map the fungal diseases GLS, NLB, and SLB noted above, we have used them to map resistance QTLs for GW and BLS (Qiu, Cooper *et al.* 2020; Qiu, Kaiser *et al.* 2020).

In this study, we examined 4 DRIL populations that share a common recurrent parent, Oh7B, to identify disease resistance QTLs to 4 foliar diseases (GW, GLS, NCLB, SCLB). The resistant donors for the 4 populations we used in this study were Ki3, NC262, NC304, and NC344. The NC lines all originate from the North Carolina State University maize breeding program and have some shared parentage (Nelson *et al.* 2016; Lopez-Zuniga *et al.* 2019). All 4 populations have been evaluated for NCLB, SCLB, and GLS previously (Lopez-Zuniga *et al.* 2019). In a previous study, we identified regions that conferred resistance to GW, BLS, NCLB, GLS, and SCLB in the NC344 × Oh7B population (Qiu, Cooper *et al.* 2020). Here, we evaluated 3 DRIL populations, with resistance donors Ki3, NC262, and NC304, for GW. We combined the existing data for SCLB, NCLB, and GLS from Lopez-Zuniga *et al.* (2019) with the newly generated GW data on the Ki3, NC262, and NC304 populations to examine resistance to bacterial and fungal diseases in maize (Supplementary Fig. 1). We adopted multiple mapping approaches to identify the genetic regions underlying MDR (Supplementary Fig. 1), which together provide an overview of the potential resistance alleles available from the parents.

The overall goal of this study was to identify regions of the genome associated with resistance to GW and to identify regions of the genome associated with resistance to multiple diseases. Our objectives were to (1) identify GW QTL within individual DRIL populations, (2) identify GW QTL across all the DRIL populations, and (3) identify regions associated with variation in MDR in each individual population using a multivariate analysis. Overall, we dissected the complicated genetic architecture of MDR in multiple populations and identified regions associated with resistance to multiple diseases that are candidates for future studies.

## Materials and methods

### Plant materials

Four maize DRIL populations first reported in Lopez-Zuniga *et al.* (2019) were included in this study: DRIL38, DRIL58, DRIL68, and DRIL78. The 4 populations share a common recurrent parent, Oh7B, which is a MDS line and have introgressions from the MDR donor lines Ki3, NC262, NC304, and NC344, respectively. The populations were created by crossing the donor parent to the recurrent parent Oh7B, followed by backcrossing for 3 generations to the recurrent parent and 4 generations of self-pollination via single seed descent to generate BC_3_F_4:5_ populations.

### Field design

Three populations (DRIL38, DRIL58, and DRIL68) were evaluated in 2018 and 2019 for GW severity at the University of Illinois Crop Sciences Research and Education Centers. The DRIL38 population was evaluated at Urbana, IL in 2018 and 2019, with 2 replications in 2018 and 2 replications in 2019. DRIL58 and DRIL68 populations were evaluated in Urbana, IL (2 replications) and Savoy, IL (2 replications) in 2019. All trials were conducted using an incomplete block design and designed using *agricolae* in the statistical software R (de Mendiburu and de Mendiburu 2020; R Core Team 2021). The 2 parental lines for each population were included in each plot as check lines. Plots were 3.2 m with 0.76 m alleys and row spacing of 0.762 m. For each plot, a total of 20 seeds for each individual line in the population were machine-planted.

### Phenotypic evaluation

Every plant in the plot was inoculated with *C. nebraskensis* isolate 16Cmn001 (10^7^ colony-forming units per milliliter) (Cooper *et al.* 2018; Mullens and Jamann 2021) . Plants were inoculated twice 1 week apart, starting when the plants were at approximately the V4 growth stage using the pin-prick method (Chang *et al.* 1977; Pataky 1985). The plants were evaluated visually to assess the necrotic area of the leaves on a per plot basis using a visual percentage rating with a 5% interval, as described in Cooper *et al.* (2018). The first rating was taken at 45 days after planting in 2019 and each plot was evaluated 3 times with an approximate 7-day interval between ratings. For the DRIL38 population planted in 2018 at Urbana, only 1 visual percentage rating was taken at 62 days after planting because there was less disease in this experiment. Because of this, throughout the analyses, these data [the DRIL38 2018 disease leaf area and 2019 area under the disease progress curve (AUDPC) values] were analyzed separately. For Md and joint linkage mapping, only the 2019 AUDPC LsMeans were used.

The number of plants in each plot was recorded, and in the later analysis, the phenotypic scores for plots with a stand count of less than 4 were coded as missing data. For the 2019 field experiments, each plot had 3 rating scores, and AUDPC values for each plot were calculated in R using the package “agricolae” (de Mendiburu and de Mendiburu 2020). Data collected in 2018 for the DRIL38 population included only 1 visual rating so no AUDPC value was calculated, and instead percentage disease leaf area ratings were used for further analysis.

### Phenotypic data analysis

LsMeans were estimated using the SAS program “proc mixed”. To calculate the LsMeans: For the single environment (DRIL38 AUDPC and diseased leaf area) datasets, we fit the model: $Y_{\mathrm{ijkl}}=\mu +G_{i}+R_{j}+B_{(j)k}+\;\epsilon_{ijkl}$

where *μ* represents the overall mean, $G_{i}$ represents the fixed genotype effect, $R_{j}$ represents the random replication effect, $B_{(j)k}$ represents the random blocking effect that nested with the replication, and $\epsilon_{ijkl}$ represents the residuals.

To calculate the LSMeans for each genotype across environments, we fit the model (the DRIL58 and DRIL68 datasets): $Y_{\mathrm{ijklm}}=\mu +E_{i}+G_{j}+R_{(i)k}+B_{(ik)l}+EG_{ij}+\epsilon_{ijklm}$.

In the combined environment model, *μ* represents the overall mean, *E_i_* represents the random environment effect, *G_j_* represents the fixed genotype effect, $R_{(i)k}$ represents the random replication effect nested within environment, $B_{(ik)l}$ represents the blocking effect nested within the replication and environment, $EG_{ij}$ represents for the interaction between environment and genotype effects, and $\epsilon_{ijkl}$ represents for the residuals.

Using the models above, lines that were statistically different (either more resistant or more susceptible) from Oh7B were identified using multiple comparison tests by performing contrasts that compared the DRIL genotype to the recurrent parent using only the values for Oh7B that were in the planting blocks for that population. Contrasts were conducted using “proc mixed” with Dunnett's adjustment, and significance was determined using a threshold of *α* = 0.05. Only data from 2019 for DRIL38 were used to identify lines that were significantly different from Oh7B.

We combined the GW data with pre-existing data for 3 other fungal foliar diseases (GLS, NCLB and, SCLB) (Lopez-Zuniga *et al.* 2019) and, using the genotypic mean estimates, calculated correlation coefficients using the “Spearman” method, a built-in function in R (Spearman 1904; R Core Team 2022).

### QTL mapping

Processed genotypic data were obtained from Lopez-Zuniga *et al.* (2019). For individual lines of interest, the number of introgressions was counted by recording the number of times that there was a stretch of more than 2 markers with the donor allele. LsMeans were used as the phenotypic data for QTL mapping. QTL mapping was conducted for each population separately using the program ICIMapping version 4.2 (Meng *et al.* 2015). ICIMapping uses a likelihood ratio test based on reducing collinearity between markers and stepwise regression to identify significant markers in populations consisting of non-idealized CSSLs (Wang *et al.* 2006, 2007). The function “CSL: QTL mapping with chromosome segment substitution lines” was used and the threshold was determined by a total of 1,000 permutations with a 0.10 type I error rate.

### Multivariate analysis

A composite statistic based on Mahalanobis distance was used to identify marker associations that represent multivariate outliers as described by Lopez-Zuniga *et al.* (2019). Outlier markers are those that do not follow the pattern of the majority of the data point cloud (Rousseeuw and Van Zomeren 1990). Each of the 4 diseases served as a variable and the Mahalanobis distance (MD) method was used to combine the 4 variates to detect outlier markers. Data for NCLB, SCLB, and GLS were obtained from Lopez-Zuniga *et al.* (2019). The MD multivariate analysis method (Qiu, Cooper *et al.* 2020) was employed for the 3 populations, namely DRIL38, DRIL58, and DRIL68. A multivariate analysis for the DRIL78 population has already been published, and thus was not conducted as part of this study (Qiu, Cooper *et al.* 2020). Mahalanobis distance was calculated based on the 4 negative log10 *P*-values of the LOD scores derived from the single-disease mapping results. Outlier markers were detected based on *P*-values for MD. To control for multiple comparisons, the false discovery rate (FDR) was calculated by adjusting the *P*-values using the “BH” method (Hochberg and Benjamini 1990) with the *p.adjust* function in R. Markers were declared to be significant using a 1% FDR.

### Joint linkage mapping

To conduct joint linkage mapping, a common set of markers genotyped across all 4 populations was required and separate from the genotypic dataset used for the individual population QTL analyses. Previously, the markers used for the individual population analyses were filtered based on each individual population and so markers that were monomorphic within 1 population were removed from that population and were kept for the other populations where that marker was polymorphic. We needed a marker dataset that included markers in common across all populations and had higher density (total marker number) than the combination of the processed marker data used for the individual QTL mapping. To accomplish this and maximize the markers included in the joint linkage mapping, we started with the raw SNP data, which was obtained from Lopez-Zuniga *et al.* (2019). First, missing data for lines were manually imputed according to the flanking markers. Imputation was conducted for up to 2 consecutive missing marker values. For each line in the population, if the 2 flanking markers were same genotype, then the missing marker type was imputed as the flanking marker; if the 2 flanking markers differed, then the missing marker was imputed as the recurrent parent (Oh7B) marker type.

Before conducting joint linkage mapping, we conducted quality control of the genotypic data using the “qtl” package in R (Broman *et al.* 2003). We imported the genotype with the cross type set to “riself”. The homozygous marker type of the female parent, Oh7B, in the 4 populations was coded as “AA”; the homozygous marker type from donor parent was coded as “BB”; and the heterozygous alleles were coded as “AB”. Quality control was conducted using the following protocol:

1. Within a DRIL population, if the 2 parental lines were monomorphic for a certain marker, then any other genotype at this marker was coded as missing (NA).
2. The percentage of missing genotypes for both lines in the populations and the markers was calculated. The lines and the markers with more than 60 missing values were dropped from the final joint stepwise regression analysis.
3. The populations were BC_3_F_4_ and thus the expected genotypes were 93.8% for AA, 4.88% for BB, and 1.38% for AB. To identify possible outcrossed lines, introgression distortion rates across all the populations were tested using chi-square tests for each line and all the markers. A Bonferroni correction with a 5% error rate was used.
4. Introgression distortion rates were then tested to identify markers that deviated from the expected introgression ratio for each population individually using a 1% error rate with a Bonferroni correction. Since the chi-square test requires that the expected value in any category be greater than 5, the AB and BB categories were examined together.

After dropping the markers and lines that failed the quality control parameters, a map with common markers for all 4 of the populations (DRIL38, DRIL58, DRIL68, and DRIL78) was created and used for the joint linkage mapping. The genotypic dataset used for joint linkage mapping can be found in Supplementary File 4.

Joint linkage mapping has greater power for QTL detection and better mapping resolution since an overall larger population is used (Buckler *et al.* 2009), and joint stepwise regression has been used to analyze families of NILs (Kolkman *et al.* 2020). For joint linkage mapping, the genotypic data were formatted as a HapMap file using TASSEL (Bradbury *et al.* 2007). There was some leftover heterozygosity and missing data that could not be resolved with the manual imputation completed as described above, so we used the “numerical” option in TASSEL5 program (version: 20191112) to impute the missing data. The “Euclidean” method with a 0.05 error rate was used for imputation. Imputation was conducted for each population separately and a joined genotypic dataset was created. The estimated combined environment LsMeans values were used as the phenotypic data, and the 4 populations were indicated as a factor in the phenotype file. The data for GW for the DRIL78 population were obtained from Qiu, Cooper *et al.* (2020). Joint stepwise regression analysis (*P* = 0.05, maximum included = 100, permutation = 1,000) was conducted for each disease separately, and the markers were nested within population.

## Results

### Disease distributions and correlations

**Fig. 1.**
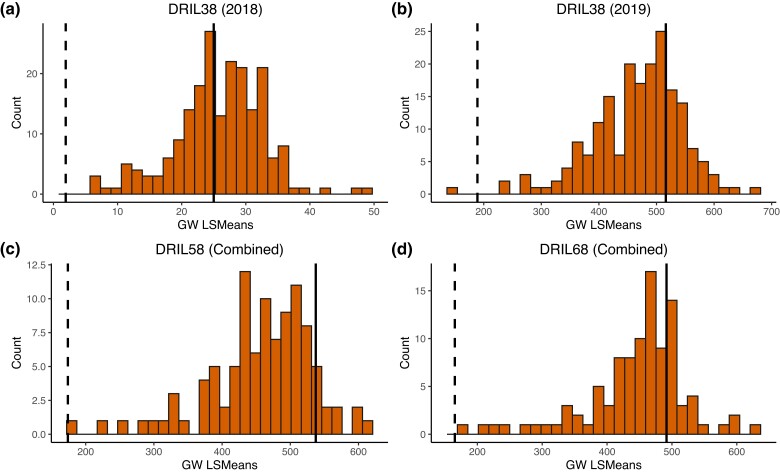
Phenotypic distribution of GW in the 3 populations. For the DRIL58 and DRIL68 populations, combined environment LsMeans estimations for AUDPC of the GW data are presented. For the DRIL38 population, there was only 1 rating in 2018 and no AUDPC calculated. Thus, the DRIL38 2018 estimation distribution was not combined with the DRIL38 2019 estimation, which was based on the AUDPC calculated using 3 ratings. The solid line indicates the LsMean of the common recurrent parent Oh7B and the dashed lines indicate the LsMeans of the donor parents, which is different for each population. The donor line for DRIL38 population is Ki3; for DRIL58 population is NC262 and for DRIL68 population is NC304.

DRIL38, DRIL58, and DRIL68 were evaluated in Urbana, IL for GW severity in 2018 and 2019. The correlations between environments were high. The Spearman correlation coefficient between environments for populations DRIL38, DRIL58, and DRIL68 was 0.47 (*P* < 0.0001), 0.80 (*P* < 0.0001), and 0.71 (*P* < 0.0001), respectively. We observed substantial variation in disease severity in all 3 DRIL populations (Fig. 1). We identified lines that were significantly more resistant to GW compared to the recurrent parent Oh7B in all populations, suggesting that GW resistance alleles derived from the donor parent were segregating in these populations. We did not observe any lines that were completely immune, nor did we observe any hypersensitive responses, which are associated with resistance genes. Lines more susceptible than the recurrent parent were also observed for all 3 populations, suggesting that the donor line also contributed alleles that conferred susceptibility. It is also possible that there were interactions between recurrent and donor parent genes that resulted in lines more susceptible than the recurrent parent.

We hypothesized that there might be a relationship between resistance to GW and the foliar fungal diseases for which these populations have been evaluated previously, NCLB, SCLB, and GLS. To test this hypothesis, we first examined the pairwise correlations among the 4 diseases in each of the 3 populations DRIL38, DRIL58, and DRIL68. The strongest correlations among diseases were in the DRIL38 population, where resistance to GW was significantly and positively correlated with resistance to all 3 fungal diseases (*ρ* = 0.25–0.36, *P* < 0.001). The strongest relationship detected was between resistance to GW and NCLB, with relatively stable correlations across the 3 populations (*ρ* = 0.26–0.36, *P* < 0.01). Resistance to GW and GLS, as well as GW and SCLB, was significantly correlated in 1 of the 3 populations (Table 1; Supplementary Fig. 2). GW and NCLB are both vascular diseases (Mullens and Jamann 2021; Chung *et al*. 2010), and, thus, are believed to have more similarities in terms of how the pathogens cause disease than GW and SCLB or GLS. GW and NCLB also have similarities in terms of pathogen lifestyle, as they both appear to have a biotroph phase, which may also contribute to the higher correlations (Kotze *et al.* 2019; Shumilak *et al.* 2023).

**Table 1.** Correlation between diseases in 3 populations.

Population	Disease			
DRIL38 (Ki3/Oh7B) *N* = 191		SCLB	NCLB	GLS
	GW	0.36***	0.33***	0.25***
	SCLB		0.30***	0.34***
	NCLB			0.45***
DRIL58 (NC262/Oh7B) *N* = 95		SCLB	NCLB	GLS
	GW	0.09	0.26**	0.19
	SCLB		0.16	0.13
	NCLB			0.20
DRIL68 (NC304/Oh7B) *N* = 99		SCLB	NCLB	GLS
	GW	0.045	0.36***	0.18
	SCLB		0.20*	0.33***
	NCLB			0.32**

Data for SCLB, NCLB, and GLS were obtained from Lopez-Zuniga *et al.* (2019).

*P < 0.05, **P < 0.01, and ***P < 0.001.

We conducted Dunnett's tests to identify lines that were significantly different than the recurrent parent for each population (Table 2; Supplementary File 1). For the DRIL38, DRIL58, and DRIL68 populations, 10, 40, and 20% of the lines were significantly more resistant to GW than Oh7B, respectively (Table 2). We also examined whether there was any overlap between lines significantly different than the recurrent parent for GW and lines significantly different for the 3 other foliar diseases. Lines that were significantly different from Oh7B for multiple diseases were identified, including 3 lines that were associated with all 4 diseases (Table 2; Supplementary File 1). The 3 lines significant using the Dunnett's tests for all diseases had at least 9 introgressions per line with introgressions in at least 3 regions that were significant in the Mahalanobis distance analysis.

**Table 2.** Dunnett's test for each population showing the number of lines that were significantly different from the recurrent parent Oh7B.

Disease	DRIL38 Ki3/Oh7B *N* = 190	DRIL58 NC262/Oh7B *N* = 101	DRIL68 NC304/Oh7B *N* = 100
SCLB	36 (36/0)	35 (35/0)	37 (37/0)
NCLB	24 (24/0)	2 (2/0)	15 (15/0)
GLS	2 (2/0)	37 (37/0)	1 (1/0)
GW	20 (19/1)	41 (41/0)	22 (19/3)
SCLB/NCLB	13	2	9
SCLB/GLS	2	14	1
SCLB/GW	9	21	11
NCLB/GLS	1	2	0
NCLB/GW	12	2	6
GLS/GW	1	19	0
SCLB/NCLB/GLS	1	2	0
SCLB/NCLB/GW	8	2	4
NCLB/GLS/GW	1	10	0
SCLB/NCLB/GLS/GW	1	2	0

For single diseases, the numbers in parentheses indicate the number of lines that are significantly more resistant than Oh7B and the number of lines that are significantly more susceptible than Oh7B, respectively. For multiple diseases, more information can be found in Supplementary File 1.

### GW QTL detection in multiple populations

QTL mapping was conducted for the 3 populations (DRIL38, DRIL58, and DRIL68) for GW resistance. There were 13 GW resistance QTLs detected across the 3 populations (Table 3). Significant markers in bins 1.02 and 7.02 were identified in more than 1 population (Table 3). The DRIL68 QTL in chromosomal bin 8.03 had the largest effect estimate of all the QTL. The DRIL68 QTL detected in chromosomal bin 3.09 explained the most phenotypic variation, with 15% of the phenotypic variation explained, but otherwise most QTL had small effects and explained less than 10% of the total phenotypic variation (Table 3). The resistant and susceptible parents contributed both resistance and susceptibility alleles, as indicated by the negative and positive additive effect estimates. While the resistant donor parent contributed most of the resistance alleles mapped in the populations, based on the additive effect estimates from the single population mapping, Ki3 and NC304 both contributed a susceptibility allele for 1 QTL in the DRIL38 and DRIL68 populations, respectively.

**Table 3.** Significant QTL detected for GW in 3 populations DRIL38, DRIL58, and DRIL68.

Peak marker	Pop.	Chr.*^a^*	cM	Pos.*^b^*	Bin*^c^*	Dataset	LOD*^d^*	Add*^e^*	PVE (%)*^f^*
*PHM13094-8*	DRIL68	1	25.64	8,348,403	1.01	Combined	8.1	−50.2	12.2
*PHM4531-46*	DRIL38	1	48.66	22,891,879	1.02	19Urbana	3.28	−21.3	5.22
	DRIL58	1	48.66	22,891,879	1.02	Combined	4.21	−39.8	11.3
*PZA00192-6*	DRIL58	1	68.84	35,583,899	1.03	Combined	3.67	−35.4	9.75
*PHM2187-46*	DRIL38	1	121.1	157,149,026	1.05	19Urbana	5.05	30.4	8.20
*PZA00289-11*	DRIL58	1	180.4	216,101,748	1.07	Combined	5.33	−43.0	14.4
*PHM2672-19*	DRIL68	3	193.1	221,520,140	3.09	Combined	9.36	49.8	14.8
*PZA00941-2*	DRIL38	4	123.6	186,659,058	4.08	19Urbana	6.5	−37.0	10.8
*PHM3691-18*	DRIL38	5	89.15	38,506,897	5.03	19Urbana	4.62	−45.4	7.47
*PZA02247-1*	DRIL38	6	64.18	146,570,902	6.05	19Urbana	4.59	−27.2	7.50
*PHM15501-6*	DRIL38	7	72.81	30,693,107	7.02	18Urbana	2.81	−3.17	6.41
	DRIL68	7	72.81	30,693,107	7.02	Combined	4.09	−30.7	5.59
*PHM2350-17*	DRIL58	8	55.4	23,964,235	8.03	Combined	2.95	−36.0	7.53
*PHM4134-8*	DRIL68	8	65.36	106,620,464	8.03	Combined	4.35	−68.3	6.09
*PHM1871-19*	DRIL68	9	60.81	28,413,009	9.03	Combined	7.16	−40.6	10.7

The combined environment datasets were used, except for the DRIL38 population, where mapping was conducted separately for each year.

^*a*^Chromosome.

^*b*^The physical position (RefGen_v3) of significant markers.

^*c*^Chromosomal bin location of significant QTL (Davis *et al*. 1999).

^*d*^LOD value at the position of the peak likelihood of the QTL. A permutation test was conducted to determine the LOD threshold for the significant markers.

^*e*^Additive effect estimates of the detected QTL. Effects are in terms of the disease rating scale used. A negative value indicates that the donor allele increased resistance.

^*f*^Percentage of the phenotypic variance explained (PVE) by the detected QTL.

In the DRIL38 population, there was an enrichment for Ki3 alleles at the significant markers (QTL) from the individual population mapping analysis, with 28% of the markers having the Ki3 allele in the Dunnett's test significant lines compared to 10% of the markers having the Ki3 allele in the lines that were not significant using the Dunnett's test. Of the 20 lines that were significant from the Dunnett's test, 3 had 3 Ki3 introgressions at the significant markers, 6 had introgressions at 2 QTLs, 9 had introgressions at 1 QTL, and 2 did not have any Ki3 introgressions at the QTL. In the DRIL68 population, there was an enrichment for introgressions from NC304 at the QTL in the lines that were significant from the Dunnett's test, where 18% of the markers had the NC304 genotype, while only 6% had the NC304 alleles in the lines that were not significant from the Dunnett's test at the 5 QTLs. Of the 22 lines that were significant in the Dunnett's test in the DRIL68 population, 1 had 3 introgressions at the QTL, 3 had 2 introgressions and the QTL, and 11 had 1 QTL, and 7 did not have any introgressions at the significant markers. In the DRIL58 population, 10 of the 41 lines had introgressions at 2 of the QTL, 15 had 1 introgression at a QTL, and 16 did not have introgressions at the QTL. These findings suggest that combining the populations and conducting joint linkage mapping might improve the power to detect QTL that were not detected in the individual populations.

### Comparisons of QTL for multiple diseases across multiple populations

Five regions were significant for more than 1 disease ∗ population combination (Table 4). These regions fell into 3 different categories—(1) significant for multiple diseases in 1 population (3 regions), (2) significant for 1 disease across multiple populations (2 regions), and (3) significant for multiple diseases across multiple populations (4 regions). Of the 5 common significant markers, 3 markers were associated with both GLS and GW, indicating that resistance to these 2 diseases is possibly linked (Table 4).

**Table 4.** Common markers identified for multiple diseases across different populations in ICI mapping analysis.

Marker	Population	Dis*^a^*	Chr.*^b^*	cM	Pos*^c^*	Bin*^d^*
PHM4531-46	DRIL38	GW	1	48.66	22,891,879	1.02
		NCLB				
		GLS				
	DRIL58	GW				
PZA00192-6	DRIL78	GLS	1	68.84	35,583,899	1.03
	DRIL58	GLS				
PHM15864-8	DRIL78	GLS	4	87.18	151,565,558	4.06
		GW				
PZA00941-2	DRIL38	NCLB	4	123.58	186,659,058	4.08
		GW				
	DRIL58	SCLB				
PHM229-15	DRIL38	GLS	9	60.82	30,087,788	9.03
	DRIL68	SCLB				

^*a*^Disease.

^*b*^Chromosome.

^*c*^The physical position (RefGen_v3) of significant markers.

^*d*^Chromosomal bin location of significant QTL (Davis *et al*. 1999).

### Identification of MDR QTL using the MD method

**Fig. 2.**
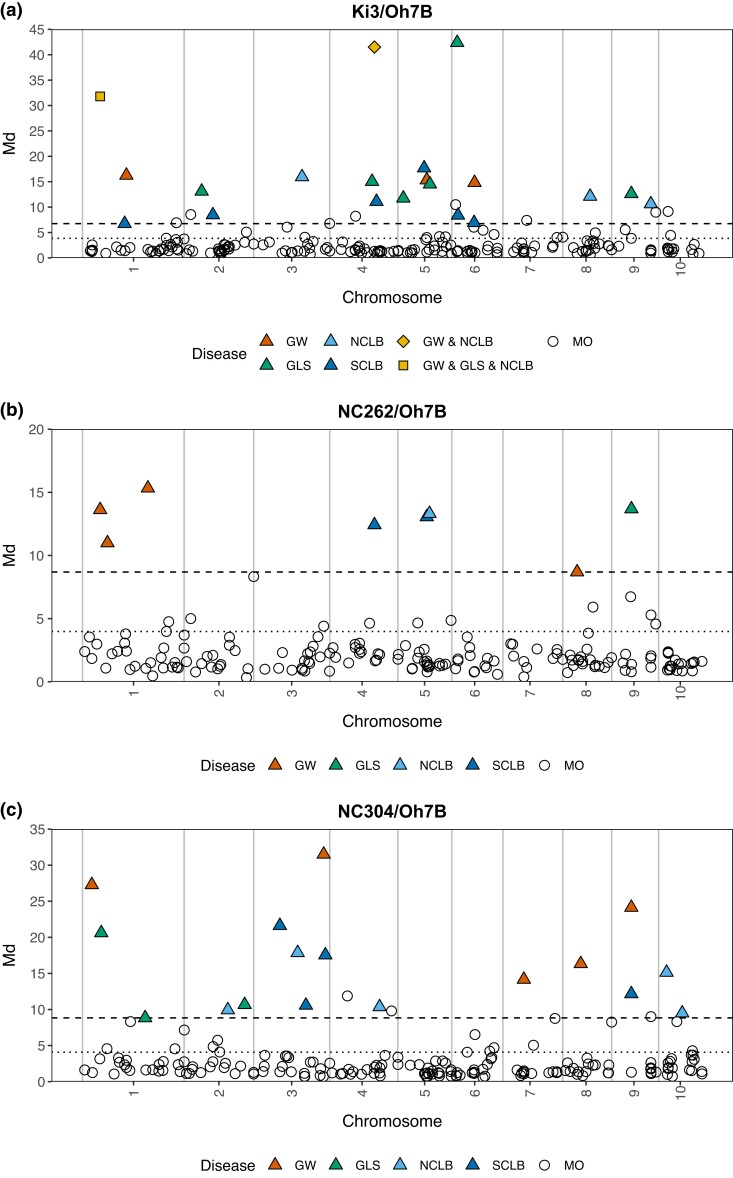
Manhattan plot for individual and multivariate analysis. The dotted line indicates the 1% FDR for the Mahalanobis distance statistic. The dashed line represents the Md value for the minimum LOD threshold for the 4 mapping analyses.

To identify QTL with effects on multiple diseases, multivariate analysis was conducted using the results of individual linkage mapping analyses. All the QTLs identified in the individual mapping analysis were significant in the multivariate analysis because a significant Mahalanobis distance value can arise due to a single trait (Fig. 2). Additionally, we were able to detect signal from markers that may not have been significant for any individual disease but are associated with an effect on more than 1 disease (Fig. 2; Supplementary File 3). Thus, this method allowed us to capture markers that have a small effect on more than 1 disease.

In the DRIL38 (Ki3/Oh7B) population, Mahalanobis distance identified 44 significant markers, of which 20 markers were identified in the single disease mapping analysis including 5 associated with resistance to GW, 7 with resistance to GLS, 5 with resistance to NCLB, and 6 with resistance to SCLB (Table 3; Supplementary File 3). The QTL on chromosome 1 (marker *PHM4531-46*) and on chromosome 4 (marker *PZA00941-2*) was detected for multiple diseases in the single disease mapping analysis (Fig. 2). We observed QTL clustering. Bin 4.08 is an interesting potential MDR region in the DRIL38 population, as this region was associated with all 4 diseases (Supplementary File 3). Bins 5.03, 6.01, and 6.05 were also associated with MDR; 3 QTLs from the multivariate analysis were identified in each of these bins and were linked to more than 1 disease according to the individual disease mapping analysis (Fig. 2; Supplementary File 3).

In the DRIL58 (NC262/Oh7B) population, 20 QTLs were identified in the multivariate analysis, of which 8 were also identified in the single disease linkage mapping analysis (Supplementary File 3). The highest number of QTLs (4) was detected for GW resistance, including 3 on chromosome 1 and 1 on chromosome 8 (Fig. 2). Bins 1.01 and 9.03 were MDR regions in the DRIL58 population since they both harbored at least 2 QTLs based on the multivariate analysis (Fig. 2).

**Fig. 3.**
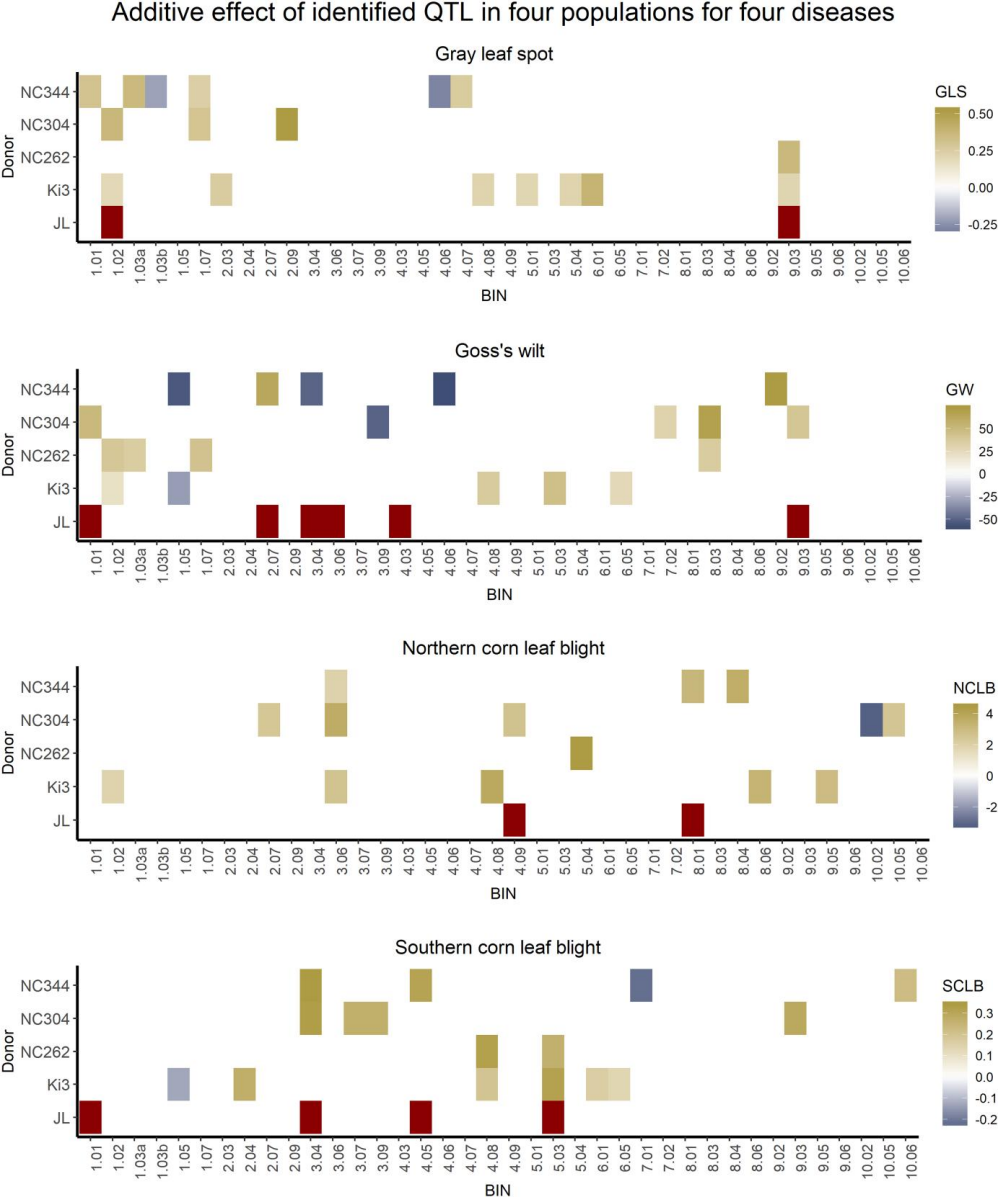
Additive effect of QTL in 4 populations for 4 diseases. The QTL detected from single disease mapping and joint linkage mapping are visualized by chromosomal bin for each disease separately. The *x*-axis indicates the chromosomal bin that the significant marker was in. The donor line of the 4 individual populations and the joint linkage mapping is indicated on the *y*-axis. The additive value of the 4 individual populations is indicated by the color of the box with positive values suggesting that donor allele increasing the resistance to the disease. For joint linkage mapping, only the presence of the QTL was shown, as no additive effects were calculated. JL indicates joint linkage mapping.

A total of 36 QTLs were identified in the DRIL68 (NC304/Oh7B) population, and 17 of those were identified in single disease mapping analysis (Supplementary File 3). Single-disease related QTLs were identified on all chromosomes except 5 and 6 (Fig. 2). Bin 3.09 and bin 9.03 were MDR regions. Both bins 3.09 and bin 9.03 were associated with resistance to GW and SCLB (Fig. 2). Although bin 3.09 is associated with resistance to GW and SCLB, the additive effect of the QTL in bin 3.09 for GW is negative while the additive value is positive for resistance to SCLB (Fig. 3). This suggests that the donor allele or linked donor alleles in this region increased susceptibility to GW but resistance to SCLB.

### Joint linkage mapping for individual diseases

Joint linkage mapping has more power to identify QTL shared by multiple populations than analyzing each population individually, but has a lower power to detect QTL that are specific to a specific population (Buckler *et al.* 2009; Ogut *et al.* 2015; Kolkman *et al.* 2020). For example, joint linkage mapping can identify QTLs when there were not enough lines with an introgression in a given region in a single population, but when combining data across populations, a QTL can be detected. We conducted joint linkage mapping to study disease resistance in 4 DRIL populations (DRIL38, DRIL58, DRIL68, and DRIL78). Joint linkage mapping was performed in TASSEL for each disease separately for the combined 4 populations. A total of 2 QTLs for resistance to GLS, 4 QTLs for resistance to SCLB, 6 QTLs for resistance to GW, and 2 QTLs for resistance to NCLB disease resistance were identified across the 4 populations through joint linkage mapping (Table 5; Fig. 3). Among these, QTLs in bin 3.04 (marker *PZA00348-11*) and in bin 9.03 (marker *PHM1871-19*) were significant for multiple diseases (Table 5). There were 2 QTLs in bin 1.01 (marker *PZA00181-2* for SCLB and *PZA00175-2* for GW) located very close to each other (∼0.2 Mb), thus, we hypothesized that those 2 QTLs might be detecting the same underlying resistance gene(s). Interestingly, this QTL in bin 1.01 is also associated with multiple diseases (Table 5).

**Table 5.** Significant markers identified in the joint linkage mapping.

Peak marker	Chr.*^a^*	cM	Physical Pos.*^b^*	BIN*^c^*	Dis*^d^*
PZA00181-2	1	25.64	8,346,760	1.01	SCLB
PZA00175-2	1	25.86	8,553,473	1.01	GW
PHM13619-5	1	47.76	22,252,464	1.02	GLS
PZA00824-2	2	117.77	197,553,232	2.07	GW
PZA00348-11	3	68.94	32,780,891	3.04	GW
					SCLB
PHM1959-26	3	105.64	170,153,721	3.06	GW
PHM259-11	4	52.47	14,374,208	4.03	GW
PHM15427-11	4	70.75	34,049,995	4.05	SCLB
PZA01332-2	4	137.84	207,440,469	4.09	NCLB
PHM565-31	5	82.66	24,398,410	5.03	SCLB
PHM2487-6	8	28.92	8,120,340	8.01	NCLB
PHM1871-19	9	60.81	28,413,009	9.03	GLS
					GW

^*a*^Chromosome.

^*b*^The physical position (RefGen_v3) of significant markers.

^*c*^Chromosomal bin location of significant QTL (Davis *et al*. 1999).

^*d*^Disease.

## Discussion

In order to protect crops from multiple biotic threats, there is a need to understand the genetic architecture of resistance to both fungal and bacterial pathogens. By combining our data for GW with previously published data for 3 fungal diseases, we were able to identify genomic regions associated with resistance to both bacterial and fungal diseases. For example, in contrast to the DRIL78 population where no lines were significantly more resistant or susceptible than Oh7B for more than 2 diseases (Qiu, Cooper *et al.* 2020), 1 line in the DRIL38 and 2 lines in the DRIL58 populations were significantly more resistant than the recurrent parent for all 4 diseases. Several regions were implicated in MDR including 1.02, 1.03, 3.04, 4.06, 4.08, and 9.03, which includes regions not previously described as regions conferring MDR in any of the DRIL populations (Lopez-Zuniga *et al.* 2019; Qiu, Cooper *et al.* 2020).

We used different mapping methods to identify disease resistance QTL across multiple populations for multiple diseases, and the results from the 3 mapping analyses were complimentary. Using multivariate analysis, we were able to identify several MDR QTLs that were not detected in individual mapping analyses. Joint linkage mapping has higher power in cases where QTLs are shared among families. Likewise, using joint linkage mapping, we were able to detect QTL not detected in individual mapping analysis, such as the SCLB resistance QTL in bin 1.01 and GW resistance QTL in bin 4.03. However, in some cases, the markers identified as significant in more than 1 population were not significant in the joint linkage mapping. For example, there was a QTL for NCLB resistance in bin 3.06, but no QTL was identified in this region in the joint linkage mapping. This is also the case for the GW resistance QTL in bin 1.02, the GLS resistance QTL in bin 1.07, and the SCLB resistance QTL in bin 4.08. One possible explanation for this could be that in our analysis, slightly different marker sets were used in the single and joint population analyses. A similar finding was reported in Singh *et al.* (2016) in that QTL were identified in individual populations but not in the joint linkage mapping.

One objective of this study was to identify QTL associated with resistance to GW in multiple populations. Between the individual population analyses and the joint linkage mapping, we identified 19 regions that were associated with GW resistance. The mapping results from this study, as well as previously published studies, all indicate that resistance is largely quantitative, as defined by Poland *et al.* (2009); (Nelson *et al.* 2018), as we observe a range of phenotypes in segregating populations that very from moderately resistant to susceptible. Based on the distributions and mapping results, there do not appear to be large-effect QTL or resistance genes for GW resistance in these populations, which is consistent with previous studies using other populations (Cooper *et al.* 2018; Cooper *et al.* 2019); however, Hu *et al.* (2018) identified a hypersensitive response associated with the rust resistance locus *rp1*. In this study, every line that was included had some disease, and no lines included in this study exhibited a hypersensitive response. Thus, resistance appears to be quantitative for GW resistance in these populations, and we do not expect that resistance genes underlie resistance in these populations.

Some of the QTL were identified in multiple DRIL populations in the individual mapping analysis, such as the QTL in bins 1.02 (marker *PHM4531-46*) and 7.02 (marker *PHM15501-6*). The significant marker *PHM4531-46* for the QTL in bin 1.02 was also detected in a RIL population developed from B73 and HP301 for GW resistance (Singh *et al.* 2016). Interestingly, this is a locus that has been shown to be involved in resistance to other diseases including Stewart's wilt (another bacterial disease) and NCLB (also a vascular disease) and a remorin gene has been implicated for disease resistance within this locus (Jamann *et al.* 2016). Additional study is needed to determine whether these previous findings relate to GW as well. Additionally, Singh *et al.* (2016) identified QTL in bins 2.07 and 9.03 for GW resistance through joint linkage mapping in populations with different parents than our study. Cooper *et al.* (2018) identified QTL for GW resistance in bins 2.07 and 7.02 in the intermated B73×Mo17 population. The overlapping of GW QTLs in bins 1.01, 1.02, 2.07, 7.02, and 9.03 in this study and previous studies suggest that these are important regions for GW resistance.

One interesting finding from this study was that there were lines that were significantly different than the recurrent parent for multiple diseases. Of the 3 lines that were significant for all 3 diseases in the Dunnett's test, DRIL38.172 has an introgression at PHM4531-46 (bin 1.02), which was associated with resistance to GW, NCLB, and GLS in the single disease mapping analysis. Interestingly, none of the other lines that have introgressions at that same region showed significant differences for all 4 diseases, indicating that it may be the stacking of other QTL that conferred MDR in that line. DRIL58.103 had an introgression at PZA00941-2 (bin 4.08) that was associated with resistance to SCLB in the DRIL58 population. All 3 of these lines would be interesting to follow up on, as well as following up on the MDR QTL at which these lines had introgressions.

From the combined results of all the mapping methods, we identified regions that were associated with multiple diseases across different populations. The QTL located in bin 9.03 (marker *PHM1871-19*) was associated with resistance to GLS, SCLB, and GW in the 4 populations. This QTL was located ∼12 Mb downstream of the MDR gene *ZmCCoAOMT2* encoding a caffeoyl-CoA O-methyltransferase (Yang *et al.* 2017), which confers resistance to SLB and GLS. A previous study identified bin 9.02–9.03 as conferring resistance to multiple diseases (Belcher *et al.* 2012). Bin 9.03 was also detected as 1 of the largest QTL for SCLB resistance in a RIL population developed from Ki14 and B73 (Zwonitzer *et al.* 2010). Bin 1.01 was another interesting QTL associated with MDR. There were 2 significant markers located very close to each other in bin 1.01 (marker *PZA00181-2* and marker *PZA00175-2* are only 206,713 bp apart) that were significant for SCLB and GW in the joint linkage mapping.

Bin 3.04 is a locus with effects on multiple diseases including resistance to GW and SCLB. The colocalization of GW and SCLB resistance at 3.04 could be due to the pleiotropic effect of a single gene associated with multiple diseases or due to the effects of multiple linked genes, each corresponding to the single disease. We do not have high enough resolution and fine-mapping is required to distinguish linkage vs pleiotropy for bin 3.04. A leucine rich repeat receptor kinase referred to as *ChSK1* underlies a SCLB resistance QTL at 3.04 and confers increased susceptibility to SCLB (Chen *et al.* 2023). Previously, bin 3.04 has been reported to confer resistance to multiple fungal diseases (Belcher *et al.* 2012; Lopez-Zuniga *et al.* 2019; Qiu, Cooper *et al.* 2020) and also to European corn borer, Fusarium stalk rot, common rust, and maize mosaic diseases (McMullen and Simcox 1995; Wisser *et al.* 2006). Bin 3.04 also harbors disease resistance gene including rust resistance gene *rp3* (Webb *et al.* 2002). Due to the low marker density in this study, it is likely that there are several smaller QTLs in bin 3.04 and each QTL may confer an effect to a different disease. Further investigation is needed.

## Conclusions

In summary, we identified regions of the genome that conferred resistance to GW and regions implicated in resistance to multiple diseases. In order to take advantage of the data that have been collected on multiple diseases and populations, we conducted 3 different types of analysis to detect GW and MDR QTL. We mapped 13 GW QTLs in the individual populations and an additional 6 using joint linkage mapping, including colocalizing QTL in bins 1.02, 7.02, and 8.03. This study confirms that resistance to GW is highly quantitative and likely not based on major gene resistance. We detected 3 lines that were resistant to all 4 diseases examined in this study. Several regions were implicated in MDR including 1.02, 1.03, 3.04, 4.06, 4.08, and 9.03. These loci could be important for breeding for MDR in maize and are targets of further study including confirmation and fine-mapping.

## Supplementary Material

jkad275_Supplementary_Data

## Data Availability

Supplemental data are available at 10.6084/m9.figshare.22532314. Supplementary File 1 has the Dunnett's test showing lines that were statistically significant than Oh7B for each disease in each population. Supplementary File 2 has the raw phenotypic data. Supplementary File 3 has the comparison of individual mapping analysis and multivariate mapping analysis for multiple diseases across multiple populations. Supplementary File 4 contains the genotypic data used for QTL mapping and joint linkage mapping. Supplemental material available at G3 online.

## References

[jkad275-B1] Bauske EC , Friskop AJ. 2021. Effects of hybrid susceptibility and inoculation timing on Goss's bacterial wilt and leaf blight severity and corn yield. Plant Dis. 105:1765–1770. doi:10.1094/PDIS-08-20-1786-RE. 33406859

[jkad275-B2] Belcher AR , Zwonitzer JC, Santa Cruz J, Krakowsky MD, Chung CL, Nelson R, Arellano C, Balint-Kurti PJ. 2012. Analysis of quantitative disease resistance to southern leaf blight and of multiple disease resistance in maize, using near-isogenic lines. Theor Appl Genet. 124(3):433–445. doi:10.1007/s00122-011-1718-1. 21997760

[jkad275-B3] Bradbury PJ , Zhang Z, Kroon DE, Casstevens TM, Ramdoss Y, Buckler ES. 2007. TASSEL: software for association mapping of complex traits in diverse samples. Bioinformatics. 23(19):2633–2635. doi:10.1093/bioinformatics/btm308. 17586829

[jkad275-B4] Broders K , Iriarte-Broders G, Bergstrom GC, Byamukama E, Chilvers M, Cruz C, Dalla-Lana F, Duray Z, Malvick D, Mueller D, et al 2022. Phyllachora species infecting maize and other grass species in the Americas represents a complex of closely related species. Ecol Evol. 12(4):e8832. doi:10.1002/ece3.8832. 35494500 PMC9036037

[jkad275-B5] Broman KW , Wu H, Sen S, Churchill GA. 2003. R/qtl: QTL mapping in experimental crosses. Bioinformatics. 19(7):889–890. doi:10.1093/bioinformatics/btg112. 12724300

[jkad275-B6] Buckler ES , Holland JB, Bradbury PJ, Acharya CB, Brown PJ, Browne C, Ersoz E, Flint-Garcia S, Garcia A, Glaubitz JC, et al 2009. The genetic architecture of maize flowering time. Science. 325(5941):714–718. doi:10.1126/science.1174276. 19661422

[jkad275-B7] Carson ML . 1991. Relationship between leaf freckles and wilt severity and yield losses in closely related maize hybrids. Phytopathology. 81(1):95–98. doi:10.1094/Phyto-81-95.

[jkad275-B8] Chang CM , Hooker AL, Lim SM. 1977. Inoculation technique for determining Stewarts bacterial leaf-blight reaction in corn. Plant Dis Rep. 61:1077–1079.

[jkad275-B9] Chen C , Zhao Y, Tabor G, Nian H, Phillips J, Wolters P, Yang Q, Balint-Kurti P. 2023. A leucine-rich repeat receptor kinase gene confers quantitative susceptibility to maize southern leaf blight. New Phytol 238(3):1182–1197. doi:10.1111/nph.18781. 36721267

[jkad275-B10] Chung CL , Longfellow JM, Walsh EK, Kerdieh Z, Van Esbroeck G, Balint-Kurti P, Nelson RJ. 2010. Resistance loci affecting distinct stages of fungal pathogenesis: use of introgression lines for QTL mapping and characterization in the maize - Setosphaeria turcicapathosystem. BMC Plant Biol. 10:103. doi:10.1186/1471-2229-10-103. 20529319 PMC3017769

[jkad275-B11] Cooper JS , Balint-Kurti PJ, Jamann TM. 2018. Identification of quantitative trait loci for Goss's wilt of maize. Crop Sci. 58(3):1192–1200. doi:10.2135/cropsci2017.10.0618.

[jkad275-B12] Cooper JS , Rice BR, Shenstone EM, Lipka AE, Jamann TM. 2019. Genome-wide analysis and prediction of resistance to Goss's wilt in maize. Plant Genome. 12(2):180045. doi:10.3835/plantgenome2018.06.0045. PMC12810136 31290921

[jkad275-B13] Davis GL , McMullen MD, Baysdorfer C, Musket T, Grant D, Staebell M, Xu G, Polacco M, Koster L, Melia-Hancock S, et al 1999. A maize map standard with sequenced core markers, grass genome reference points and 932 expressed sequence tagged sites (ESTs) in a 1736-locus map. Genetics. 152:1137–1172. doi:10.1093/genetics/152.3.1137. 10388831 PMC1460676

[jkad275-B14] de Mendiburu F , Yaseen M. agricolae: Statistical Procedures for Agricultural Research. R package version 1.4.0. https://myaseen208.github.io/agricolae/https://cran.r-project.org/package=agricolae.2020.

[jkad275-B15] Gou M , Balint-Kurti P, Xu M, Yang Q. 2023. Quantitative disease resistance: multifaceted players in plant defense. J Integr Plant Biol. 65:594–610. doi:10.1111/jipb.13419. 36448658

[jkad275-B16] Hao Y , Hu Y, Jaqueth J, Lin J, He C, Lin G, Zhao M, Ren J, Tamang TM, Park S, et al 2023. Genetic and transcriptomic dissection of host defense to Goss's bacterial wilt and leaf blight of maize. G3 (Bethesda). 13:jkad197. doi:10.1093/g3journal/jkad197. 37652038 PMC10627284

[jkad275-B17] Harding MW , Jindal K, Tambong JT, Daayf F, Howard RJ, Derksen H, Reid LM, Tenuta AU, Feng J. 2018. Goss's bacterial wilt and leaf blight of corn in Canada—disease update. Can J Plant Pathol. 40:471–480. doi:10.1080/07060661.2018.1506502.

[jkad275-B18] Hochberg Y , Benjamini Y. 1990. More powerful procedures for multiple significance testing. Stat Med. 9:811–818. doi:10.1002/sim.4780090710. 2218183

[jkad275-B19] Hu Y , Ren J, Peng Z, Umana AA, Le H, Le H, Danilova T, Fu J, Wang H, Robertson A, et al 2018. Analysis of extreme phenotype bulk copy number variation (XP-CNV) identified the association of rp1 with resistance to Goss's wilt of maize. Front Plant Sci. 9:110. doi:10.3389/fpls.2018.00110. 29479358 PMC5812337

[jkad275-B20] Jackson TA , Harveson RM, Vidaver AK. 2007. Reemergence of Goss's wilt and blight of corn to the central High Plains. Plant Management Network. doi:10.1094/PHP-2007-0919-01-BR.

[jkad275-B21] Jamann TM , Luo X, Morales L, Kolkman JM, Chung CL, Nelson RJ. 2016. A remorin gene is implicated in quantitative disease resistance in maize. Theor Appl Genet. 129:591–602. doi:10.1007/s00122-015-2650-6. 26849237

[jkad275-B22] Jindal KK , Zhu X, Woldemariam T, Tenuta AU, Jindal M, Javed N, Daayf F, Reid LM. 2019. Maize inbreds for multiple resistance breeding against major foliar, ear and stalk rot diseases. Maydica. 64:1–22.

[jkad275-B23] Kolkman JM , Strable J, Harline K, Kroon DE, Wiesner-Hanks T, Bradbury PJ, Nelson RJ. 2020. Maize introgression library provides evidence for the involvement of liguleless1 in resistance to northern leaf blight. G3 (Bethesda). 10:3611–3622. doi:10.1534/g3.120.401500. 32816917 PMC7534436

[jkad275-B24] Kotze RG , van der Merwe CF, Crampton BG, Kritzinger Q. 2019. A histological assessment of the infection strategy of *Exserohilum turcicum* in maize. Plant Pathol. 68:504–512. doi:10.1111/ppa.12961.

[jkad275-B25] Li W , Liao C-J, Bluhm BH, Mengiste T, Woloshuk CP. 2022. A maize (*Zea mays* L.) BIK1-like receptor-like cytoplasmic kinase contributes to disease resistance. Plant Mol Biol Rep. 40:28–42. doi:10.1007/s11105-021-01299-2.

[jkad275-B26] Lopez-Zuniga LO , Wolters P, Davis S, Weldekidan T, Kolkman JM, Nelson R, Hooda KS, Rucker E, Thomason W, Wisser R, et al 2019. Using maize chromosome segment substitution line populations for the identification of loci associated with multiple disease resistance. G3 (Bethesda). 9:189–201. doi:10.1534/g3.118.200866. 30459178 PMC6325898

[jkad275-B27] Martins LB , Rucker E, Thomason W, Wisser RJ, Holland JB, Balint-Kurti P. 2019. Validation and characterization of maize multiple disease resistance QTL. G3 (Bethesda). 9:2905–2912. doi:10.1534/g3.119.400195. 31300480 PMC6723135

[jkad275-B28] McMullen MD , Simcox KD. 1995. Genomic organization of disease and insect resistance genes in maize. Mol Plant Microbe Interact. 8:811–815. doi:10.1094/MPMI-8-0811.

[jkad275-B29] Mehl KM , Mikel MA, Bradley CA. 2021. Evaluation of corn germplasm accessions for resistance to *Clavibacter nebraskensis*, causal agent of Goss's bacterial wilt and leaf blight. Plant Dis. 105:156–163. doi:10.1094/PDIS-11-19-2394-RE. 33118875

[jkad275-B30] Mehl KM , Weems JD, Ames KA, Bradley CA. 2015. Evaluation of foliar-applied copper hydroxide and citric acid for control of Goss's wilt and leaf blight of corn. Can J Plant Pathol. 37:160–164. doi:10.1080/07060661.2015.1012741.

[jkad275-B31] Meng L , Li H, Zhang L, Wang J. 2015. QTL IciMapping: integrated software for genetic linkage map construction and quantitative trait locus mapping in biparental populations. Crop J. 3:269–283. doi:10.1016/j.cj.2015.01.001.

[jkad275-B32] Miedaner T , Juroszek P. 2021. Global warming and increasing maize cultivation demand comprehensive efforts in disease and insect resistance breeding in north-western Europe. Plant Pathol. 70:1032–1046. doi:10.1111/ppa.13365.

[jkad275-B33] Mueller DS , Wise KA, Sisson AJ, Allen TW, Bergstrom GC, Bissonnette KM, Bradley CA, Byamukama E, Chilvers MI, Collins AA, et al 2020. Corn yield loss estimates due to diseases in the United States and Ontario, Canada, from 2016 to 2019. Plant Health Prog. 21:238–247. doi:10.1094/PHP-05-20-0038-RS.

[jkad275-B34] Mueller DS , Wise KA, Sisson AJ, Allen TW, Bergstrom GC, Bosley DB, Bradley CA, Broders KD, Byamukama E, Chilvers MaI, et al 2016. Corn yield loss estimates due to diseases in the United States and Ontario, Canada from 2012 to 2015. Plant Health Prog. 17:211–222. doi:10.1094/PHP-RS-16-0030.

[jkad275-B35] Mullens A , Jamann TM. 2021. Colonization and movement of green fluorescent protein-labeled *Clavibacter nebraskensis* in maize. Plant Dis. 105(5):1422–1431. doi:10.1094/PDIS-08-20-1823-RE. 33190611

[jkad275-B36] Nelson PT , Krakowsky MD, Coles ND, Holland JB, Bubeck DM, Smith JSC, Goodman MM. 2016. Genetic characterization of the North Carolina State University maize lines. Crop Sci. 56:259–275. doi:10.2135/cropsci2015.09.0532.

[jkad275-B37] Nelson R , Wiesner-Hanks T, Wisser R, Balint-Kurti P. 2018. Navigating complexity to breed disease-resistant crops. Nat Rev Genet. 19:21–33. doi:10.1038/nrg.2017.82. 29109524

[jkad275-B38] Nene YL . 1988. Multiple-disease resistance in grain legumes. Annu Rev Phytopathol. 26:203–217. doi:10.1146/annurev.py.26.090188.001223.

[jkad275-B39] Ogut F , Bian Y, Bradbury PJ, Holland JB. 2015. Joint-multiple family linkage analysis predicts within-family variation better than single-family analysis of the maize nested association mapping population. Heredity (Edinb). 114:552–563. doi:10.1038/hdy.2014.123. 25585918 PMC4434247

[jkad275-B40] Osdaghi E , Robertson AE, Jackson-Ziems TA, Abachi H, Li X, Harveson RM. 2023. *Clavibacter nebraskensis* causing Goss's wilt of maize: five decades of detaining the enemy in the New World. Mol Plant Pathol. 24:675–692. doi:10.1111/mpp.13268. 36116105 PMC10257048

[jkad275-B41] Owusu V , Mira M, Soliman A, Adam LR, Daayf F, Hill RD, Stasolla C. 2019. Suppression of the maize phytoglobin ZmPgb1.1 promotes plant tolerance against *Clavibacter nebraskensis*. Planta. 250:1803–1818. doi:10.1007/s00425-019-03263-7. 31456046

[jkad275-B42] Pataky JK . 1985. Relationships among reactions of sweet corn hybrids to Goss's wilt, Stewart’s bacterial wilt, and northern corn leaf blight. Plant Dis. 69:845–848. doi:10.1094/PD-69-845.

[jkad275-B43] Pataky JK . 1988. Classification of sweet corn hybrid reactions to common rust, northern leaf blight, Stewart’s wilt, and Goss’ wilt and associated yield reductions. Phytopathology. 78:172–178. doi:10.1094/Phyto-78-172.

[jkad275-B44] Poland JA , Balint-Kurti PJ, Wisser RJ, Pratt RC, Nelson RJ. 2009. Shades of gray: the world of quantitative disease resistance. Trends Plant Sci. 14:21–29. doi:10.1016/j.tplants.2008.10.006. 19062327

[jkad275-B45] Qiu Y , Cooper J, Kaiser C, Wisser R, Mideros SX, Jamann TM. 2020. Identification of loci that confer resistance to bacterial and fungal diseases of maize. G3 (Bethesda). 10:2819–2828. doi:10.1534/g3.120.401104. 32571803 PMC7407448

[jkad275-B46] Qiu YT , Kaiser C, Schmidt C, Broders K, Robertson AE, Jamann TM. 2020. Identification of quantitative trait loci associated with maize resistance to bacterial leaf streak. Crop Sci. 60:226–237. doi:10.1002/csc2.20099.

[jkad275-B47] R Core Team . 2021. R: A Language and Environment for Statistical Computing. Vienna: R Foundation for Statistical Computing.

[jkad275-B48] R Core Team . 2022. R Core Team. A Language and Environment for Statistical Computing. Vienna: R Foundation for Statistical Computing. pp. ISBN 3–900051–07–0. http://www.R-project.org/.

[jkad275-B49] Rousseeuw PJ , Van Zomeren BC. 1990. Unmasking multivariate outliers and leverage points. J Am Stat Assoc. 85:633–639. doi:10.1080/01621459.1990.10474920.

[jkad275-B50] Savary S , Willocquet L, Pethybridge SJ, Esker P, McRoberts N, Nelson A. 2019. The global burden of pathogens and pests on major food crops. Nat Ecol Evol. 3:430–439. doi:10.1038/s41559-018-0793-y. 30718852

[jkad275-B51] Schaefer CM , Bernardo R. 2013. Genomewide association mapping of flowering time, kernel composition, and disease resistance in historical Minnesota maize inbreds. Crop Sci. 53:2518–2529. doi:10.2135/cropsci2013.02.0121.

[jkad275-B52] Shumilak A , El-Shetehy M, Soliman A, Tambong JT, Daayf F. 2023. Goss's wilt resistance in corn is mediated via salicylic acid and programmed cell death but not jasmonic acid pathways. Plants. 12:1475. doi:10.3390/plants12071475. 37050101 PMC10097360

[jkad275-B53] Singh A , Andersen AP, Jackson-Ziems TA, Lorenz AJ. 2016. Mapping quantitative trait loci for resistance to Goss's bacterial wilt and leaf blight in North American maize by joint linkage analysis. Crop Sci. 56:2306–2313. doi:10.2135/cropsci2015.09.0543.

[jkad275-B54] Singh A , Li G, Brohammer AB, Jarquin D, Hirsch CN, Alfano JR, Lorenz AJ. 2019. Genome-wide association and gene co-expression network analyses reveal complex genetics of resistance to Goss's wilt of maize. G3 (Bethesda). 9:3139–3152. doi:10.1534/g3.119.400347. 31362973 PMC6778796

[jkad275-B55] Singh AK , Singh VK, Singh A, Ellur RK, Pandian RTP, Gopala Krishnan S, Singh UD, Nagarajan M, Vinod KK, Prabhu KV. 2015. Introgression of multiple disease resistance into a maintainer of Basmati rice CMS line by marker assisted backcross breeding. Euphytica. 203:97–107. doi:10.1007/s10681-014-1267-1.

[jkad275-B56] Spearman C . 1904. The proof and measurement of association between two things. Am J Psychol. 15:72–101. doi:10.2307/1412159. 3322052

[jkad275-B57] Vidaver AK , Mandel M. 1974. *Corynebacterium nebraskense*, a new, orange-pigmented phytopathogenic species. Int J Syst Bacteriol. 24:482–485. doi:10.1099/00207713-24-4-482.

[jkad275-B58] Wang H , Hou J, Ye P, Hu L, Huang J, Dai Z, Zhang B, Dai S, Que J, Min H, et al 2021. A teosinte-derived allele of a MYB transcription repressor confers multiple disease resistance in maize. Mol Plant. 14:1846–1863. doi:10.1016/j.molp.2021.07.008. 34271176

[jkad275-B59] Wang J , Wan X, Crossa J, Crouch J, Weng J, Zhai H, Wan J. 2006. QTL mapping of grain length in rice (*Oryza sativa* L.) using chromosome segment substitution lines. Genet Res. 88:93–104. doi:10.1017/S0016672306008408. 17125584

[jkad275-B60] Wang J , Wan X, Li H, Pfeiffer WH, Crouch J, Wan J. 2007. Application of identified QTL-marker associations in rice quality improvement through a design-breeding approach. Theor Appl Genet. 115:87–100. doi:10.1007/s00122-007-0545-x. 17479243

[jkad275-B61] Webb CA , Richter TE, Collins NC, Nicolas M, Trick HN, Pryor T, Hulbert SH. 2002. Genetic and molecular characterization of the maize rp3 rust resistance locus. Genetics. 162:381–394. doi:10.1093/genetics/162.1.381. 12242248 PMC1462242

[jkad275-B62] Webster BT , Curland RD, Hirsch CD, McNally RR, Malvick DK, Ishimaru CA. 2020. Genetic diversity and phylogeny of strains of *Clavibacter nebraskensis* associated with recent and historic Goss's wilt epidemics in the north central USA. Plant Pathol. 69:990–1002. doi:10.1111/ppa.13185.

[jkad275-B63] Wiesner-Hanks T , Nelson R. 2016. Multiple disease resistance in plants. Annu Rev Phytopathol. 54:229–252. doi:10.1146/annurev-phyto-080615-100037. 27296142

[jkad275-B64] Wisser RJ , Balint-Kurti PJ, Nelson RJ. 2006. The genetic architecture of disease resistance in maize: a synthesis of published studies. Phytopathology. 96:120–129. doi:10.1094/PHYTO-96-0120. 18943914

[jkad275-B65] Wisser RJ , Kolkman JM, Patzoldt ME, Holland JB, Yu J, Krakowsky M, Nelson RJ, Balint-Kurti PJ. 2011. Multivariate analysis of maize disease resistances suggests a pleiotropic genetic basis and implicates a GST gene. Proc Natl Acad Sci U S A. 108:7339–7344. doi:10.1073/pnas.1011739108. 21490302 PMC3088610

[jkad275-B66] Yang Q , He Y, Kabahuma M, Chaya T, Kelly A, Borrego E, Bian Y, El Kasmi F, Yang L, Teixeira P, et al 2017. A gene encoding maize caffeoyl-CoA O-methyltransferase confers quantitative resistance to multiple pathogens. Nat Genet. 49:1364–1372. doi:10.1038/ng.3919. 28740263

[jkad275-B67] Yu H , Ruan H, Xia X, Chicowski AS, Whitham SA, Li Z, Wang G, Liu W. 2022. Maize FERONIA-like receptor genes are involved in the response of multiple disease resistance in maize. Mol Plant Pathol. 23:1331–1345. doi:10.1111/mpp.13232. 35596601 PMC9366073

[jkad275-B68] Zwonitzer JC , Coles ND, Krakowsky MD, Arellano C, Holland JB, McMullen MD, Pratt RC, Balint-Kurti Peter J. 2010. Mapping resistance quantitative trait loci for three foliar diseases in a maize recombinant inbred line population—evidence for multiple disease resistance? Phytopathology. 100:72–79. doi:10.1094/PHYTO-100-1-0072. 19968551

