# Supplementary material for: Identification of loci conferring resistance to 4 foliar diseases of maize
Source: G3 (Bethesda). 2023 Dec 5;14(2):jkad275. doi: 10.1093/g3journal/jkad275 (PMC10849323; doi:10.1093/g3journal/jkad275)
Supplement: jkad275_Supplementary_Data [file jkad275_supplementary_data.zip › Supplemental_Figures_G3-2023-404220.pdf]

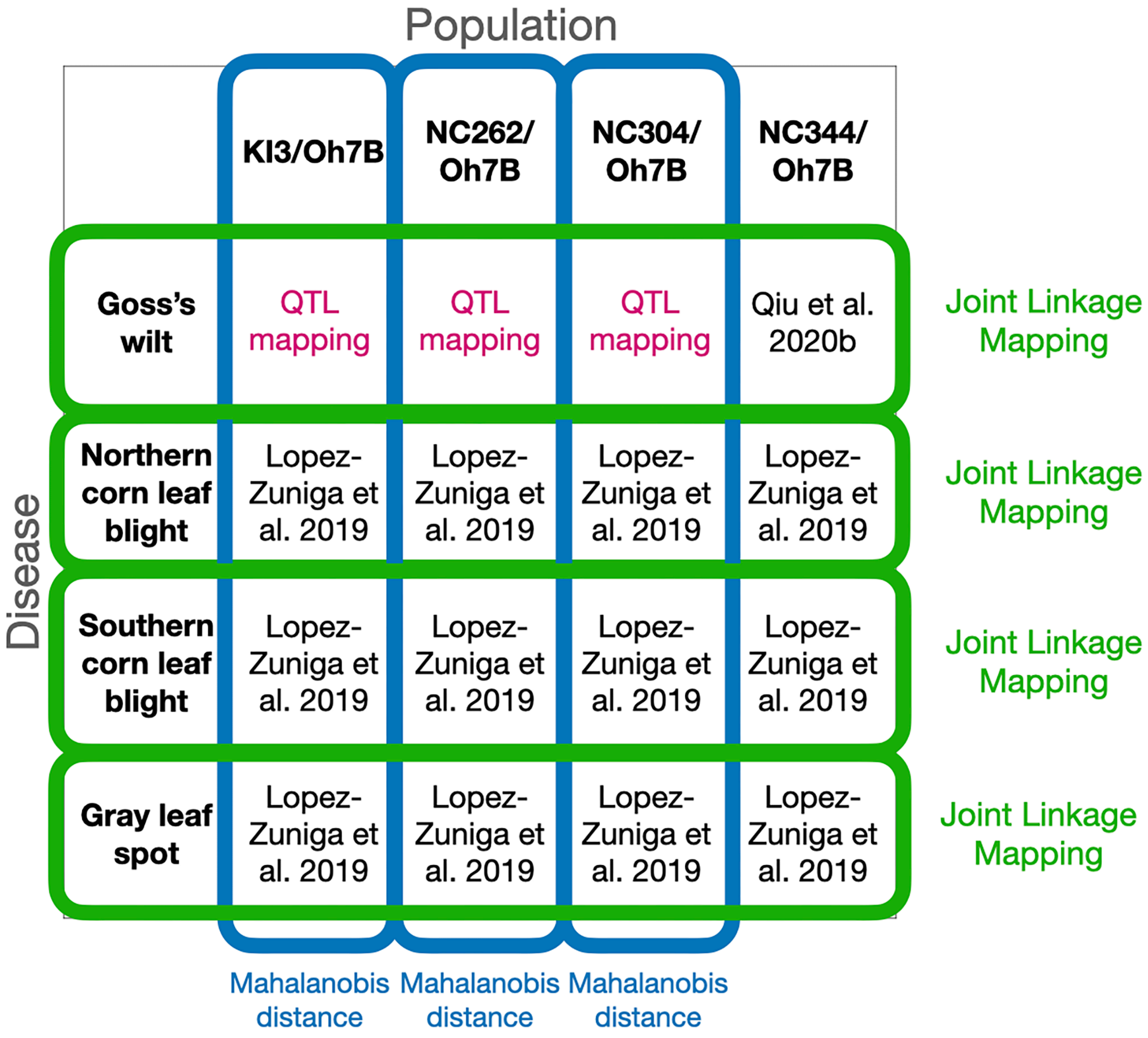

Population
Disease
KI3/Oh7B
NC262/ Oh7B
NC304/ Oh7B
NC344/ Oh7B
Goss's wilt
QTL mapping
QTL mapping
QTL mapping
Qiu et al. 2020b
Joint Linkage Mapping
Northern corn leaf blight
Lopez-Zuniga et al. 2019
Lopez-Zuniga et al. 2019
Lopez-Zuniga et al. 2019
Lopez-Zuniga et al. 2019
Joint Linkage Mapping
Southern corn leaf blight
Lopez-Zuniga et al. 2019
Lopez-Zuniga et al. 2019
Lopez-Zuniga et al. 2019
Lopez-Zuniga et al. 2019
Joint Linkage Mapping
Gray leaf spot
Lopez-Zuniga et al. 2019
Lopez-Zuniga et al. 2019
Lopez-Zuniga et al. 2019
Lopez-Zuniga et al. 2019
Joint Linkage Mapping
Mahalanobis distance
Mahalanobis distance
Mahalanobis distance

Figure S1

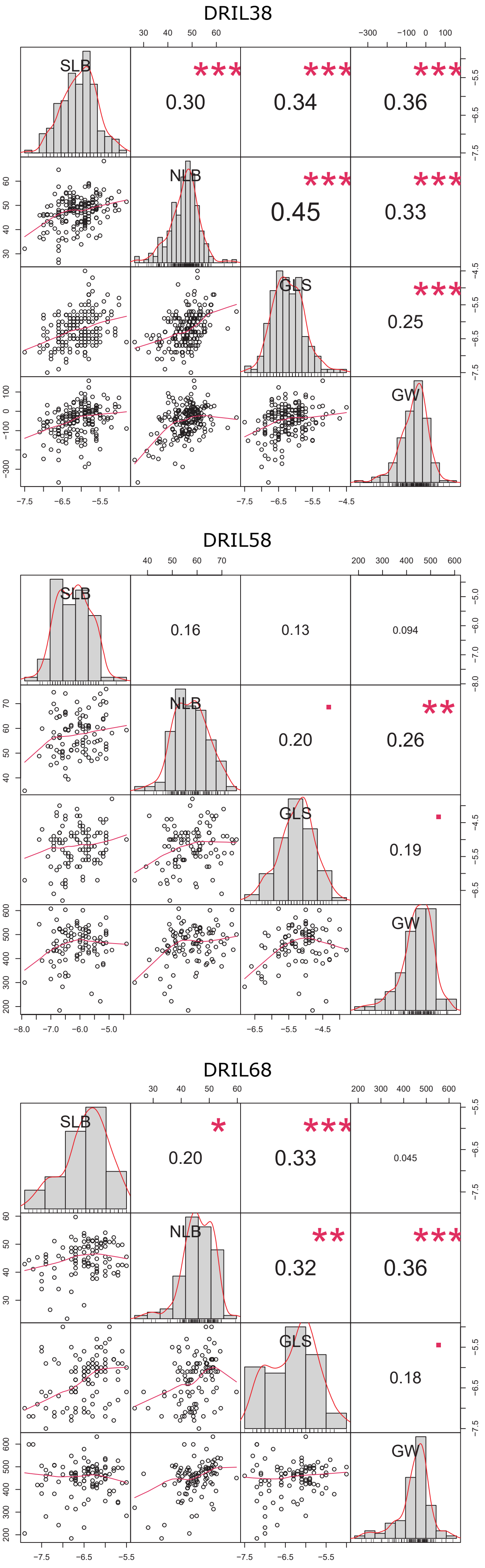
DRIL38
SLB
NLB
GLS
GW
*** 0.30
*** 0.34
*** 0.36
*** 0.45
*** 0.33
*** 0.25
DRIL58
SLB
NLB
GLS
GW
0.16
0.13
0.094
0.20
** 0.26
0.19
DRIL68
SLB
NLB
GLS
GW
* 0.20
*** 0.33
0.045
** 0.32
*** 0.36
0.18

Figure S2
