# Supplementary material for: Identification of loci conferring resistance to 4 foliar diseases of maize
Source: G3 (Bethesda). 2023 Dec 5;14(2):jkad275. doi: 10.1093/g3journal/jkad275 (PMC10849323; doi:10.1093/g3journal/jkad275)
Supplement: jkad275_Supplementary_Data [file jkad275_supplementary_data.zip › Supplemental_Material_Legends_G3-2023-404220.pdf]

Supplementary Files

File S1: Dunnett's test showing lines that were statistically significant than Oh7B for each disease in each population. Column names are explained at the bottom of the table.

File S2: Raw phenotypic data. The raw phenotypic data for the lines included in this study. Legend sheet explains column headers.

File S3: Analysis files for the individual populations, which includes the marker effect estimates for each disease for each marker, the LOD scores for each marker, and the raw MD results. Legend sheet explains column headers.

File S4: Genotypic datasets used for mapping. The first three tabs are the input genotypic datasets used in ICIMapping. The Joint map sheet is the input genotypic data for all four populations that served as the genotypic dataset input into TASSEL and is in hapmap format. The columns with the parental alleles are retained in this version for reference.

Figure S1: Datasets that have already been published (disease and population combinations) are listed with the appropriate reference. New analyses conducted as part of this manuscript are listed in pink, green and blue. The individual population analyses are in pink, the Mahalanobis distance analyses are shown in blue (across diseases, but within a single population), and the joint stepwise regression is in green (within one disease, but across populations).

Figure S2: Correlations among diseases. Correlations among diseases are shown for each population individually. The symbol ■ denotes P-value < 0.1, * P-value < 0.05, ** P-value < 0.01, and *** P-value < 0.001
